# Effects of the total replacement of fish-based diet with plant-based diet on the hepatic transcriptome of two European sea bass (*Dicentrarchus labrax*) half-sibfamilies showing different growth rates with the plant-based diet

**DOI:** 10.1186/1471-2164-12-522

**Published:** 2011-10-23

**Authors:** Florian Geay, Serena Ferraresso, Jose L Zambonino-Infante, Luca Bargelloni, Claire Quentel, Marc Vandeputte, Sachi Kaushik, Chantal L Cahu, David Mazurais

**Affiliations:** 1 Ifremer, UMR 1067, Departement Physiologie Fonctionnelle des Organismes Marins, Technopôle Brest-Iroise, BP 70, 29280 Plouzané, France; 2 Department of Public Health, Comparative Pathology, and Veterinary Hygiene, Faculty of Veterinary Medicine, University of Padova, Vialedell'Università 16, 35020 Legnaro, Italy; 3 Anses, Laboratoire de Ploufragan/Plouzané, Agence nationale de sécurité sanitaire de l'aliment, de l'environnement et du travail, Technopôle Brest-Iroise, 29 280 Plouzané, France; 4 Ifremer, chemin de Maguelone, 34250 Palavas les Flots, France; 5 INRA, UMR 1313 Génétique Animale et Biologie Intégrative, Domaine de Vilvert, 78350 Jouy-en-Josas, France; 6 INRA-UMR Nutrition Aquaculture Génomique, Pôle Hydrobiologie, 64310 Saint Pée-sur-Nivelle, France

## Abstract

**Background:**

Efforts towards utilisation of diets without fish meal (FM) or fish oil (FO) in finfish aquaculture have been being made for more than two decades. Metabolic responses to substitution of fishery products have been shown to impact growth performance and immune system of fish as well as their subsequent nutritional value, particularly in marine fish species, which exhibit low capacity for biosynthesis of long-chain poly-unsaturated fatty acids (LC-PUFA). The main objective of the present study was to analyse the effects of a plant-based diet on the hepatic transcriptome of European sea bass (*Dicentrarchus labrax*).

**Results:**

We report the first results obtained using a transcriptomic approach on the liver of two half-sibfamilies of the European sea bass that exhibit similar growth rates when fed a fish-based diet (FD), but significantly different growth rates when fed an all-plant diet (VD). Overall gene expression was analysed using oligo DNA microarrays (GPL9663). Statistical analysis identified 582 unique annotated genes differentially expressed between groups of fish fed the two diets, 199 genes regulated by genetic factors, and 72 genes that exhibited diet-family interactions. The expression of several genes involved in the LC-PUFA and cholesterol biosynthetic pathways was found to be up-regulated in fish fed VD, suggesting a stimulation of the lipogenic pathways. No significant diet-family interaction for the regulation of LC-PUFA biosynthesis pathways could be detected by microarray analysis. This result was in agreement with LC-PUFA profiles, which were found to be similar in the flesh of the two half-sibfamilies. In addition, the combination of our transcriptomic data with an analysis of plasmatic immune parameters revealed a stimulation of complement activity associated with an immunodeficiency in the fish fed VD, and different inflammatory status between the two half-sibfamilies. Biological processes related to protein catabolism, amino acid transaminations, RNA splicing and blood coagulation were also found to be regulated by diet, while the expression of genes involved in protein and ATP synthesis differed between the half-sibfamilies.

**Conclusions:**

Overall, the combined gene expression, compositional and biochemical studies demonstrated a large panel of metabolic and physiological effects induced by total substitution of both FM and FO in the diets of European sea bass and revealed physiological characteristics associated with the two half-sibfamilies.

## Background

For the majority of intensively-reared finfish species including the European sea bass (*Dicentrarchus labrax*), diets have traditionally been based on fish meal (FM) and fish oil (FO). However, the decline in worldwide supplies of marine oils and fish meal [1] has led the industry and several research initiatives to investigate the possibility of using plant proteins and vegetable oils as alternatives to marine fishery-derived proteins and oils. Nevertheless, the use of such plant products is recognised to have several disadvantages, particularly related to their protein contents, amino acid profiles and unsaturated fatty acid imbalances, but also including endogenous anti-nutritional factors. Taking into account these limits and the dietary needs of different fish species, efforts have been made over the last decade to develop diets with a low content in fish resources. This has been done by using a mixture of vegetable meals and oils [2], resulting in the successful reduction of both FM and FO in the feeds for several species [3]. Much progress has indeed been made in the substitution of FM and FO with plant products in feeds for salmonids as well as marine fish, in the recent past [4-6]. While several studies performed on salmonids indicate that total replacement of fish meal by plant ingredients leads to decreased growth rate [7,8], Kaushik et al. [9] showed that it was possible to almost totally replace fish meal with a mixture of plant protein sources for European sea bass without reducing growth performance. The same authors did, however, note a significant increase in fat content and a decrease in plasma cholesterol concentrations for sea bass fed with plant protein, suggesting altered regulation of lipid metabolic pathways.

For the replacement of fish oil, it is well established that freshwater or anadromous fish species such as salmonids have higher tolerance to vegetable oil compared with marine fish species. Thus, for Atlantic salmon (*Salmo salar*) and rainbow trout (*Oncorhynchus mykiss*), the total replacement of fish oil with a blend of vegetable oils poor in highly unsaturated fatty acids (LC-PUFA) did not result in diminished growth performance, feed conversion or development of histopathology, despite an increase of polyunsaturated fatty acid (PUFA) deposition in liver and muscle [10-12]. In some studies, a high or total substitution of fish oil by linseed and soybean oils for several months induced decreases in growth rate of gilthead sea bream (*Sparus aurata*) and European sea bass (*Dicentrarchus labrax*) [13-15]. Some other studies undertaken with gilthead sea bream showed that while there were no differences in growth of fish fed high levels of vegetable oil mixtures, there were possibly other metabolic consequences [5,16]. This lower adaptation of marine fish species to vegetable oil has been suggested to be linked to their lower efficiency at synthesizing LC-PUFA from n-3 and n-6 precursors present in plants [17-19]. A recent study performed on European sea bass indicates that the limiting step for LC-PUFA synthesis could be linked to a deficiency in the stimulation of delta-6-desaturase (FADS2) activity in fish fed vegetable oil [13]. The resulting low tissue levels of LC-PUFA in marine fish fed vegetable oil could impact fish health, since LC-PUFA are not only important as structural components of cell membranes but also as precursors of eicosanoids. Eicosanoids are involved in many physiological processes, including osmoregulation, immune responses, blood coagulation and reproduction [20-23]. Moreover, lowered eicosapentaenoic acid (EPA; 20:5n-3) and docosahexaenoic acid (DHA; 22:6n-3) content in the flesh of marine fish fed a vegetable diet diminishes their nutritional value for consumers.

Recent studies on salmonids have suggested there is genetic variability for ability to utilize plant-based diets [24-26]. Interestingly, some genotype-diet interactions for growth have also been recently demonstrated in European sea bass fed on a plant-based diet [27]. The existence of such interactions suggests that it could be possible to select fish, and particularly sea bass, with a better ability to grow on plant-based feeds. However, the genetic factors and related metabolic or physiological pathways responsible for these advantageous capacities are still unknown.

To our knowledge, studies on the total replacement of both FM and FO have not been undertaken in a marine fish species until now, except for the afore mentioned work by Le Boucher et al. (2010) [27]. Moreover, investigations on the impact of FM and FO substitution with plant products for marine fish species have only been performed using molecular and/or biochemical approaches focused on selected target metabolic pathways or physiological functions. Such dedicated approaches do not allow an exhaustive and global overview of the molecular mechanisms underlying tissue and organism response to diet substitution.

In order to gain a fuller picture of the effects of total substitution of both FM and FO, the present study primarily aimed to characterise the regulation of the liver transcriptome in European sea bass fed on a fish-free diet for 9 months, using an oligonucleotide microarray recently developed for this species [28]. This investigation was performed on liver because this organ plays a key role in intermediary metabolism, integrates a large part of nutrient uptake and affects a wide range of functions in the body, including plasma protein synthesis, hormone production and detoxification. The present study was undertaken using two half-sibfamilies that exhibited similar growth rates when they were fed a FM-FO diet, but different growth rates when they were fed a plant protein-vegetable oil based diet. The second aim of this work was to pinpoint genes and related metabolic and physiological pathways that could explain the different adaptation of these two half-sibfamilies of European sea bass to a plant-based diet. The hepatic transcriptomes and flesh LC-PUFA profiles were, therefore, compared between these half-sibfamilies.

## Methods

### Diets and fish

Two practical iso-energetic and iso-nitrogenous diets were formulated (Table 1). The first, a fish-based diet (FD), was composed of fish meal, wheat gluten and fish oil whereas the second, a vegetable-based diet (VD), was devoid of ingredients of fish origin and composed of plant protein sources and vegetable oil (linseed). The fatty acid composition of the two diets is given in Table 2.

**Table 1:** Ingredients, amino acid profiles and chemical composition of the two diets fed to European sea bass.

Diets	FD	VD
*Ingredients*		

Fish meal	38.0	0.0
Corn gluten	18.0	20.0
Soybean meal	0.0	18.2
Wheat gluten	7.2	20.0
Whole wheat	25.3	7.2
White sweet lupin	0.0	14.0
Fish oil	8.5	0.0
Linseed oil	0.0	9.4
Soy lecithin	0.0	1.0
L-lysine	0.0	2.7
Dicalcium phosphate	0.0	3.0
Binder (Sodium alginate)	1.0	1.0
Attractant mix^1^	1.0	1.5
Mineral premix^2^	1.0	1.0
Vitamin premix^3^	1.0	1.0
		
*Chemical composition*		

Dry matter (DM), g/100 g	94.5	90.3
Crude protein, g/100 g DM	49.8	50.3
Crude fat, g/100 g DM	14.3	14.1
Gross energy (GE), kJ/g DM	22.8	21.9
Ash, g/100 g DM	6.3	7.9
		
*Amino acid composition (g/100 g)*		

Arginine	2.2	1.8
Histidine	1.0	0.9
Isoleucine	1.9	1.8
Leucine	4.2	3.9
Lysine	2.5	3.6
Methionine+Cystine	1.8	1.4
Phenylalanine+Tyrosine	3.8	3.7
Threonine	1.7	1.3
Tryptophan	0.4	0.3
Valine	2.3	1.9
Glycine	2.5	3.5
Serine	2.2	4.7
Glutamic acid	10.6	22.1
Aspartic acid	3.3	7.6
Proline	2.8	5.4
Alanine	2.8	3.0

Ingredient composition (g 100 g^-1^), amino acid profiles (g 100 g^-1^) and chemical composition (g/100 g DM^-1^) of the two diets fed to European sea bass.

^1^Attractant mix contained: glycine (0.2), alanine (0.2), betaine (0.3), taurine (0.3) and glucosamine (0.5)

^2 3 ^as per NRC [75]

**Table 2:** Fatty acid composition (% sum of fatty acids) of the two diets FD and VD

Diets	FD	VD
*Fatty acids composition *		

Σ saturates	27.67	11.82
Σ monoenes	36.27	23.85
18:2n-6	8.90	23.59
20:2n-6	0.25	0.11
18:3n-6	0.22	0.10
20:4n-6	0.71	0.00
Σ n-6 PUFA	10.31	23.91
18:3n-3	1.27	40.90
18:4n-3	1.84	0.00
20:3n-3	0.13	0.07
20:4n-3	0.87	0.00
20:5n-3	9.54	0.05
22:5n-3	1.56	0.00
22:6n-3	10.52	0.12
Σ n-3 PUFA	25.73	41.14
total lipid (%)	13.80	13.10

Σ n-3 PUFA/Σ n-6 PUFA	2.5	1.7
EPA/DHA	0.9	1.0
EPA/ARA	13.6	-

All procedures concerning the animals and their handling were conducted in accordance with the Code of Ethics of the World Medical Association (Declaration of Helsinki). The study was performed under licence no. 29.021 of the French Department of Veterinary Services (Direction Départementale des Services Vétérinaires) to conduct experimental protocols and samplings on fish. The present study focused on fish of two half-sibfamilies (half-sibfamily *G *and half-sibfamily *g*), which exhibited a similar daily growth coefficient (DGC) when they were fed on a fish-based diet, but had significantly different DGCs when they were fed an all-plant diet. The two half-sibfamilies of fish were produced from a crossing design between 8 females and 41 males, which was carried out with European sea bass individuals from a Mediterranean stock held at the Experimental Station of Palavas-les-Flots (Ifremer, France) [27]. Rearing conditions have already been described by Le Boucher et *al*. [27]: fish were reared in two tanks per diet condition, supplied with recirculated seawater (38 ppt) at a constant temperature of 21°C, and subjected to a photoperiod of 12 h light:12 h dark. Fish were fed on a commercial diet (Neogrower, Le Gouessant, Lamballe, France) until they reached the mean weight of 192 g. Before the beginning of the nutritional challenge, fish were individually tagged, genotyped for microsatellite markers to infer parentage [27], and distributed randomly into two tanks per dietary condition (FD or VD), with 196 fish per tank. After an acclimation period of 2 weeks, fish were hand fed to satiation (1 meal/day) for a period of 9 months on the experimental diets (FD or VD). Analysis of experimental data was done using the following formulae:

$$\begin{array}{c} \text{Hepatosomatic index}\left(\%\right):\text{HSI}=100\times \left(\text{liver weight}\left(\text{g}\right)\times \text{body weight}{\left(\text{g}\right)}^{-1}\right)\\ \text{Viscerosomatic index}\left(\%\right):\text{VSI}=100\times \left(\text{carcass weight}\left(\text{g}\right)\times \text{body weight}{\left(\text{g}\right)}^{-1}\right) \end{array}$$

Daily growth coefficient (% day): DGC = 100 × (final individual weight (g) ^1/3 ^- initial individual weight (g) ^1/3^)/days (According to Cho [29]).

Feed efficiency (FE): [final weight (g) - initial weight (g)]/feed ration (g) (According to Carter et al. [30])

At the end of the growth trial, liver, muscle and plasma were sampled from 15 fishes per half-sibfamily and per dietary treatment. Muscular LC-PUFA profiles and real-time PCR investigations were performed on these 15 sampled individuals per group. Microarray analysis of hepatic RNA and investigation of immune parameters in plasma were investigated on 5 to 8 sampled individuals per group in order to respect similar sex ratio, sampling time and RNA quality (RNA Integrity Number as determined by Bioanalyser (Agilent) > 9).

### Lipid extraction and fatty acid analysis

One gram of white muscle was dissected from 15 fish per dietary treatment and immediately frozen in liquid nitrogen. Total lipids were extracted according to the method of Folch et *al*. [31], by Accelerated Solvent Extraction 200 (ASE, Dionex) with dichloromethane/methanol (2:1) containing 0.01% butylatedhydroxutoluene (BHT) as antioxidant. Lipids were extracted at 100 bars, 100°C, with a 5 min precallingphase, 2 min static phase, and 60% flush for 60 sec (3 cycles). The separation of neutral and polar lipids was performed according to the procedure described by Juaneda and Roquelin [32]. The total lipid (TL) extracts were fractionated on silica cartridges (Sep-Pack, Waters^®^), neutral lipids (NL) were eluted with chloroform and polar lipids (PL) with methanol. Fatty acid methyl esters (FAME) were prepared from total lipids by acid-catalysed trans-esterification. FAME were quantified by gas-liquid chromatography (Clarus 500, Perkin Elmer) with a BPX70 column of 30 m length and 0.22 mm I.D. Hydrogen was used as carrier gas and temperature programming was from 50°C to 180°C at 20°C/min and then to 220°C at 3°C/min. Individual methyl esters were identified by comparison with known standards. The fatty acid analysis was performed on one sample per fish.

### RNA extraction and real-time quantitative PCR analysis

Total mRNA of liver was extracted using Trizol reagent (Invitrogen, USA) and quantified by measuring absorbance at 260 nm in a spectrophotometer (Nanodrop Labtech, France). The reverse transcription was performed using the QuantiTect^® ^Reverse Transcription kit (QIAGEN), including a genomic DNA elimination reaction. Reactions were carried out in a volume of 20 μl, containing 1 μg of total RNA, 1 unit of Quantiscript Reverse Transcriptase, 4 μl of Quantiscript RT buffer (5 ×) and 1 μM primer mix.

Seven genes involved in metabolic and/or immune pathways of interest (*fads2, hmgcr, fabp7, angptl3, cxcl10, gck *and *lpl*), whose the oligonucleotides were spotted on the chip, were analysed by real-time PCR in order to validate the gene expression patterns obtained through the microarray approach. The relative mRNA levels were automatically normalized with housekeeping elongation factor 1 (*ef1*) gene expression and measured by Bio-Rad IQ5 software using ΔΔCt method (Ct for Cycle threshold):

Gene Normalized Expression in sample 1 = (Gene Relative Quantity in sample 1)/(EF1 Relative Quantity in sample 1) with Relative Quantity in sample 1 for a gene *i *= E_(gene *i*) _^Ct (sample 2) - Ct (sample 1)^

Ef1 was chosen to provide an internal control for real-time PCR, since contrary to 18S rRNA and actin initially tested, we did not observe any significant difference (t-student test, p > 0. 1) between Ct values for Ef1 between the dietary groups (See additional file 1: Comparison of Ct values for *ef1 *gene between the dietary groups). Its stability was also assessed by a low coefficient of variation over all the samples (CV < 5%).

Specific primers (Table 3) were designed from European sea bass sequences of *fads2 *(GenBank: EU439924), *fabp7 *(EMBL: FM000669), *hmgcr *(EMBL: CB043825), *angptl3 *(EMBL: FM023639), *cxcl10 *(EMBL: FM015474), *gck *(EMBL: AM986860), *lpl *(EMBL: DT044526) and *ef1 *(GenBank: AJ866727) (Table 3). All primers used for real-time quantitative PCR analysis were defined with the Primer3 software http://frodo.wi.mit.edu/primer3/ in order to respect an annealing temperature of 60°C. All PCR reactions were performed with an efficiency of 100% (± 5%). The PCR reactions were carried out in an I-cycler with an optical module (Bio-Rad, Hercules, CA, USA), in a final volume of 15 μl containing 7.5 μl SYBR Green Supermix (Biorad, Hercules, CA, USA), 0.5 μl of each primer (10 mM) and 5 μl of cDNA (1/10 dilution). The PCR program consisted of an initial DNA denaturation of 94°C for 90 s, followed by 45 cycles at 95°C for 30 s and 60°C for 60 s. A triplicate of the amplification reaction was realised for each sample.

**Table 3:** Primers used for each gene expression analysis by real-time PCR.

	Forward primers (5'-3')	Reverse primers (5'-3')	amplicon size
FADS2	CCTTCACTGCTCTTCATCCCAA	CCCAGGTGGAGGCAGAAGAA	202
FABP7	GAAGGCACTTGGTGTTGGTT	CAGGGTTTTCACCACCACTT	102
HMGCR	CCAGCTTCGTATTCAGCACA	GCTTTGGAGAGGTCGATGAG	105
LPL	AGTTCCACATCCGGAAACTG	GCTCCGGTTGTCTTCTTTTG	142
GCK	GGTGAAGCAAGCCTGAACTC	CTTCCAGCAGTGACTGTCCA	122
ANGPTL3	CAACATCTTGCAGGAGCGTA	CTCTCCGACAGTCCCTTCAG	77
CXCL10	GGAGAGTGAGCCAGAACCTG	CCCTTGTGCACTGAAGACAA	91
EF1	GCTTCGAGGAAATCACCAAG	CAACCTTCCATCCCTTGAAC	153

### Plasma lysozyme concentration

Plasma lysozyme activity was determined at ambient temperature using a turbidimetric assay [33], adapted to microtitration plates. Briefly, a bacterial suspension of *Micrococcus lysodeikticus *(Sigma) was prepared at a concentration of 1.25 g.l^-1 ^in a 0.05 M sodium-phosphate buffer, pH 6.2. Fifty microlitres of the samples were placed in 96-well microtitration plates. The reaction was initiated in a Labsystems-iEMS analyser, by addition of 160 μl well^-1^*M. Lysodeikticus *suspension using an automatic dispenser. The optic density (OD) reading was taken at a wavelength of 450 nm every 15 s for 3 min, the plate being shaken before each reading. Lysozyme values for samples were converted to mg.ml^-1^, using a reference curve established with hen egg white lysozyme (Sigma).

### Plasma alternative complement pathway activity

Determination of the alternative pathway of plasma complement activity was carried out at 4°C through a haemolytic assay with rabbitred blood cells (RRC, Biomérieux, Craponne, France) as described by Yano [34], adapted to microtitration plates. Sea bass samples, diluted to 1/64 in EGTA-Mg-GVB buffer to avoid natural haemolytic activity, were added in increasing amounts, from 10 to 100 μl well^-1^. Wells were filled with EGTA-Mg-GVB buffer to a final volume of 100 μl. Finally, 50 μl of 2% RRC (Biomérieux) suspension was added to each of the wells. Control values of 0% and 100% haemolysis were obtained using 100 μl of EGTA-Mg-GVB buffer and 100 μl of non-decomplemented trout haemolytic serum at 1/50 in ultrapure water, respectively. The samples were then incubated for 1 h at 20°C. The microplates were centrifuged (400 g, 5 min, 4°C) and 75 μl of supernatant from each well was then transferred into another 96-well microplate with 75 μl of phosphate buffered saline (PBS, Biomérieux). The absorbance (*A*_540_) was read in a Labsystems-iEMS analyser and the number of ACH_50 _units per ml of plasma was determined by reference to the 50% haemolysis.

### *Dicentrarchus labrax *oligonucleotide microarray

Gene expression profiles were investigated using the Agilent-019810 *Dicentrarchus labrax *oligo microarray (GEO accession: GPL9663). This platform represents 19, 035 unique transcripts of the European sea bass. Two non-overlapping probes were designed for each transcript for a total 38, 070 oligonucleotide probes (60 mers) synthesized onto the array (for details see [35]). All sequences (DLPD) are publicly available in a dedicated database [35], together with associated annotations, GO entries and putative homologous genes in fish model species.

Microarrays were synthesized *in situ *using Agilent non-contact ink-jet technology with a 4 × 44 K format, and included default positive and negative controls. Microarray analysis was based on a single color (Cy3) design. A mixture of 10 different viral polyadenilated RNAs (Agilent Spike-In Mix) was also added to each RNA sample to monitor labelling and hybridization quality as well as microarray analysis work-flow.

Sample labelling and hybridization were performed as reported in Ferraresso et *al*. [28]. Briefly, for each sample, 200 ng of total RNA were linearly amplified and labelled with Cy3-dCTP according to the Agilent One-Color Microarray-Based Gene Expression Analysis protocol. A mixture of 10 different viral polyadenilated RNAs (Agilent Spike-In Mix) was also added to each RNA sample to monitor labelling and hybridization quality as well as microarray analysis work-flow. After fragmentation, a total of 1, 650 ng of labelled cRNA were dispensed in the gasket slide and assembled to the microarray slide (each slide containing four arrays). Slides were incubated for 17 h at 65°C in an Agilent Hybridization Oven and washed following manufacturer's instructions.

### Data acquisition and normalization

Hybridized slides were scanned at 5 μm resolution using an Agilent G2565BA DNA microarray scanner. Default settings were modified to scan the same slide twice at two different sensitivity levels (XDR Hi 100% and XDR Lo 10%). The two linked images generated were analyzed together; data were extracted and background subtracted using the standard procedures contained in the Agilent Feature Extraction (FE) Software version 9.5.1. Spike-in probe intensities were used to assess the performance of the normalization procedure for each dataset. Data normalization was performed using R statistical software http://www.r-project.org; microarray data were normalized across all arrays using the cyclic loess approach [36]. Fold changes (FC) were calculated for each gene by finding the average value for each group (dietary or sibfamily group). Raw and normalized fluorescence data of the present microarray experiment have been deposited in the GEO database under accession number (*under submission: NCBI tracking system #16023742*).

### Statistical analysis

All the results are presented as mean values with standard deviations (SD). Daily Growth Coefficient (DGC) was studied using a model accounting for diet as a fixed effect and tank, sire, dam, sire*diet and dam*diet as random effects, using SAS-GLM. Effects of diet and half-sibfamily factors on biometry (hepatosomatic index: HSI and viscerosomatic index: VSI), fatty acid composition, gene expression (qPCR), plasma lysozyme concentration and alternative complement pathway activity were tested by two-way ANOVAs (*P *< 0.05) using Statistica biosoft 8.0. The microarray data were analysed by two-way ANOVA using Tmev (Tigr MultiExperiment Viewer) statistical software, and gene expression was considered significantly different when *P *< 0.01. Significant enrichment of GO biological process categories were tested for using EASE software (version 2.0) with *P *< 0.05 [35].

## Results

### Growth and biometry

After 9 months of the feeding trial, European sea bass fed VD exhibited significantly lower DGC than those FD (Table 4). In addition, the fish of half-sibfamily *G *fed the VD had a significantly higher DGC than fish of half-sibfamily *g *fed VD, while there was no difference between these two half-sibfamilies when they were both fed FD (Table 4). The hepatosomatic index (HSI) was regulated by diet and genetic factors while the viscerosomatic index (VSI) was only regulated by the genetic factor. There were no significant interactions between dietary and genetic factors for these two parameters. The feed efficiency (FE) in the duplicate tanks was 0.56 and 0.60 for the FD and 0.51 and 0.55 for the VD, respectively. Mortality was not significantly different between dietary treatment and half-sibfamilies (Table 4).

**Table 4:** Growth and biometric parameters of two half-sibfamilies of European sea bass (*G *and *g*) fed fish-based and plant-based diets.

	FD		VD				
					
	HSF g	HSF G	HSF g	HSF G	Diet factor	Sib family factor	Diet × Sib family factors
					
					*p *value	*p *value	*p *value
Initial length (cm)	22.5 ± 1.8	21.7 ± 2.5	21.2 ± 1.7	21.9 ± 1.5	NS	NS	NS
Initial weight (g)	218 ± 55	178 ± 61	179 ± 45	178 ± 36	0.01	NS	0.01
Final length (cm)	31.1 ± 2.1	30.3 ± 2.7	28.9 ± 1.8	30.0 ± 1.9	0.01	NS	NS
Final weight (g)	625 ± 138	553 ± 154	475 ± 97	513 ± 96	0.01	NS	0.01
HSI (%)	2.67 ± 0.1	2.07 ± 0.1	2.09 ± 0.1	1.90 ± 0.1	0.01	0.01	NS
VSI (%)	6.86 ± 0.24	5.44 ± 0.35	7.17 ± 0.22	6.39 ± 0.34	NS	0.01	NS
DGC^(10-4)^	103.5 ± 5.7	103.9 ± 11.1	92.2 ± 3.4	98.0 ± 6.7	0.01	NS	0.01
FE	0.56 - 0.60		0.51 - 0.55		-	-	-
Survival (%)	99.5	99.5	100	100	NS	NS	NS

Initial and final weights and lengths (n = 15), HSI (n = 75) and VSI (n = 75). Effects of diet factor and genetic factor on the biometric parameters are determined by two-way ANOVA. Results are expressed as mean +/- S.D. and significant differences are indicated by the *p *value (two-way ANOVA, *P *< 0.05).

### Lipid and fatty acid compositions of the fillet

The flesh lipid composition was significantly affected by dietary treatment (See additional file 2: Fatty acid composition in muscle). Feeding VD significantly increased the percentage of saturated lipids in both the neutral lipids and phospholipids. The α-linolenic acid (18:3n-3) and linoleic acid (18:2n-6) contents were respectively 10-fold and 3-fold higher, in both lipid classes, when fish were fed VD. In addition, AA, EPA and DHA contents were around 2.5-fold lower in flesh of fish fed VD. The Σn-3 PUFA/Σn-6 PUFA ratio decreased in both the neutral lipid (2-fold) and phospholipid (4-fold) fractions in the flesh of European sea bass fed VD.

### Microarray gene expression profiling

A list of 4, 272 significant probes was obtained for the effect of diet factor, corresponding to 582 unique transcripts with gene ontology (GO) annotation. Among these regulated transcripts, 358 exhibited higher levels in fish fed VD while 224 were over-expressed in the liver of fish fed FD. For the family factor effect, total of 989 significant probes were revealed corresponding to 199 unique transcripts with GO annotation. Among these, 116 exhibited higher levels in half-sibfamily *G *while 83 were more abundant in half-sibfamily *g*. In addition, 297 probes related to 72 genes with functional annotation exhibited significant diet × genetic interactions. The main biological processes enriched out of those associated with genes that were over-expressed in fish fed VD were physiological process (324 genes), metabolism (267 genes), RNA splicing (16 genes), protein catabolism (34 genes), aerobic respiration (5 genes), sterol metabolism (8 genes), carboxylic metabolism (30 genes), amino acid metabolism (18 genes), blood coagulation (12 genes) and hexose catabolism (8 genes) (See additional file 3: Significantly enriched biological processes associated with genes regulated by diet and half-sibfamily factors). The genes involved in carboxylic acid and sterol metabolism included fatty acid desaturase 2, steroyl-CoA desaturase 9, NADH-cytochrome b5 reductase, Isopentenyl-diphosphate delta-isomerase 1, Lanosterol 14-alpha demethylase, Farnesyl pyrophosphate synthase, C-4 methylsterol oxidase and 3-hydroxy-3-methylglutaryl-coenzyme A reductase. Apolipoprotein A-I, Apolipoprotein B-100 and lipoprotein lipase, implicated in lipid transport, were also found to be up-expressed in fish fed VD (Table 5). Similarly, some genes involved in carbohydrate metabolism (hexose catabolism), such as glucose-6-phosphate 1-dehydrogenase, 6-phosphogluconate dehydrogenase and fructose-1, 6-bisphosphatase 1 were also expressed at a higher level in the fish fed VD (Table 5). Expression levels of genes involved in protein metabolism and amino acid metabolism were also increased in fish fed VD (Table 5).

**Table 5:** Genes involved in the main physiological processes regulated by dietary treatments.

Physiological process	Swiss prot description	Gene name	Fold-change (FC)
Lipid metabolism and transport	Fatty acid desaturase 2	FADS2	4.9
	Stearoyl-CoA 9-desaturase	SCD9	1.4
	NADH-cytochrome b5 reductase 2	CYB5R2	2.0
	1-acyl-sn-glycerol-3-phosphate acyltransferase gamma	AGPAT3	2.3
	Phosphatidylserine decarboxylase proenzyme	PISD	2.8
	Phosphatidylinositol-glycan biosynthesis class F protein	PIGF	1.6
	Isopentenyl-diphosphate Delta-isomerase 1	IDI1	2.0
	Lanosterol 14-alpha demethylase	CYP51A1	3.6
	Farnesyl pyrophosphate synthase	FDPS	2.6
	C-4 methylsterol oxidase	SC4MOL	2.6
	3-hydroxy-3-methylglutaryl-coenzyme A reductase	HMGCR	4.4
	Apolipoprotein A-I	APOA1	1.3
	Apolipoprotein B-100	APOB100	1.5
	Lipoprotein lipase	LPL	3.1
	Angiopoietin-related protein 3	ANGPTL3	0.3
	Phosphatidylcholine-sterol acyltransferase	LCAT	1.9

Carbohydrate metabolism	Glucose-6-phosphate 1-dehydrogenase	G6PDH	1.3
	Hexose-6-phosphate 1-dehydrogenase	H6PDH	1.2
	6-phosphogluconate dehydrogenase	PGD	1.5
	Fructose-1, 6-bisphosphatase 1	FBP1	2.3
	Fructose-bisphosphate aldolase A	ALDOA	2.7
	Fructose-bisphosphate aldolase B	ALDOB	2.3

Protein metabolism	Proteasome subunit alpha type-4	PSMA	1.2
	Proteasome subunit beta type-7	PSMB7	1.3
	26S protease regulatory subunit 7	PSMC2	1.3
	26S proteasome non-ATPase regulatory subunit 4	PSMD4	1.5
	Ubiquitin-associated protein 1	UBAP1	1.8
	Ubiquitin-conjugating enzyme E2 A	UBE2A	1.9
	Ubiquitin-conjugating enzyme E2 G1	UBE2G1	1.7
	Ubiquitin-conjugating enzyme E2 N	UBE2N	1.2

Amino acid metabolism	CTP synthase 1	CTPS	1.7
	Glutamine amidotransferase	GMPS	1.8
	Alpha-aminoadipate aminotransferase	AADAT	1.7
	Glutamate oxaloacetate transaminase 1	GOT1	4.4
	Tyrosine aminotransferase	TAT	2.6
	Succinate dehydrogenase iron-sulfur subunit	SDHB	1.4
	Isocitrate dehydrogenase subunit gamma	IDH3g	1.3
	Malate dehydrogenase	MDH	2.5

Immune function	Interleukin-8	IL8	0.5
	C-X-C motif chemokine 10	CXCL10	0.5
	C-reactive protein	CRP	0.5
	Lysozyme g like protein	LYG	0.7
	Integrin beta-2	ITGB2	0.6
	Receptor-type tyrosine-protein phosphatase F	PTPRF	0.5
	Prostaglandin synthase 2	PTGS2	0.6
	Fatty acid-binding protein	FABP7	4.5
	Plasma protease C1 inhibitor	SERPING1	1.3
	Prostaglandin D2 synthase 2	PTGS3	0.6

Cell communication	Cytokine receptor common subunit gamma	IL2RG	0.5
	protein tyrosine phosphatase, receptor type, F	PTPRF	0.5
	Integrin beta-2	ITGB2	0.6

Blood coagulation	Antithrombin-III	SERPINC1	1.9
	Plasma protease C1 inhibitor	SERPING1	1.3
	Vitamin K-dependent protein S	PROS1	1.4
	Plasminogen	PLG	1.4
	Platelet glycoprotein 4	CD36	2.2
	Coagulation factor X	F10	1.3
	Prothrombin	F2	1.2
	Coagulation factor VII	F7	1.5
	Fibrinogen beta chain	FGB	1.3
	Fibrinogen gamma chain	FGG	1.3

The fold-changes (FC) are indicated considering FD as the reference group. (two-way ANOVA, *P *< 0.01).

In contrast, the main biological processes enriched associated with the genes that were lower-expressed in fish fed VD were particularly related to cellular process (136 genes), cell communication (65 genes) and cell proliferation (32 genes) (See additional file 3: Significantly enriched biological processes associated with genes regulated by diet and half-sibfamily factors). In addition, some genes involved in the immune function were less expressed in fish fed VD (Table 5).

The comparison of hepatic transcriptomes between the two half-sibfamilies indicated that genes involved in immune function, such as complement component C2, C3 and C9 and mannan-binding lectin serine protease 2, were more greatly expressed in half-sibfamily *g *than in half-sibfamily *G *(Table 6). Inversely, expression levels of NADH dehydrogenase genes (*ndufb4, ndufb6, ndufs4, ndufs6 *and *ndusv2*), involved in the electron transport to the respiratory chain, were significantly lower in half-sibfamily *g *compared with half-sibfamily *G*. Half-sibfamily *g *was also characterised by lower expression level of genes implicated in ATP production (*atp5c1 *and *atp5g3*) and protein synthesis, such as ribosomal subunits (*mrpl22, mrpl27, mrpl30, mrpl34, mrpl48, mrps14 *and *mrps17*) than the half-sibfamily *G *(Table 6). Among the 72 genes exhibiting an interaction between half-sibfamily and diet factors, 50 were involved in metabolism. However, only the processes related to aromatic amino acid family (*hpd, hpgd *and *hsd17b4*) and nucleotide metabolism (*ctps, dck, gmpr, nt5e *and *tln1*) were found to be over-represented among these genes (Table 7).

**Table 6:** Genes involved in the main physiological processes regulated by the genetic factor.

Physiological process	Swiss prot description	Gene name	Fold-change (FC)
Immune function	Complement C2	C2	1.2
	Complement C3	C3	1.7
	Complement component C9	C9	1.5
	Mannan-binding lectin serine protease 2	MASP2	1.3
	Tumor necrosis factor receptor superfamily member 14	TNFRS14	1.7

Electron transport for ATP synthesis	NADH dehydrogenase [ubiquinone] 1 beta subcomplex subunit 4	NDUFB4	0.8
	NADH dehydrogenase [ubiquinone] 1 beta subcomplex subunit 6	NDUFB6	0.8
	NADH dehydrogenase [ubiquinone] iron-sulfur protein 4	NDUFS4	0.8
	NADH dehydrogenase [ubiquinone] iron-sulfur protein 6	NDUFS6	0.7
	NADH dehydrogenase [ubiquinone] flavoprotein 2	NDUFV2	0.8
	Cytochrome b-c1 complex subunit 7	UQCRB	0.8

Energy pathway	ATP synthase subunit gamma	ATP5C1	0.4
	ATP synthase lipid-binding protein	ATP5G3	0.5
	Cytochrome c oxidase copper chaperone	COX17	0.8
	Cytochrome b-245 heavy chain	Cybb	0.6
	3-hydroxyisobutyrate dehydrogenase	HIBADH	0.7
	Mitochondrial inner membrane protein	OXA1L	0.8

Protein biosynthesis	T-complex protein 1 subunit beta	CCT2	0.8
	Eukaryotic translation initiation factor 4 gamma 1	EIF4G1	0.6
	Basic helix-loop-helix domain-containing protein KIAA2018	KIAA2018	0.9
	39S ribosomal protein L22	MRPL22	0.8
	39S ribosomal protein L27	MRPL27	0.8
	39S ribosomal protein L30	MRPL30	0.8
	39S ribosomal protein L34	MRPL34	0.7
	39S ribosomal protein L48	MRPL48	0.7
	28S ribosomal protein S14	MRPS14	0.8
	28S ribosomal protein S17	MRPS17	0.8
	60S ribosomal protein L18	RPL18	0.7
	60S acidic ribosomal protein P1	RPLP1	0.8
	40S ribosomal protein S18	RPS18	0.8

The fold-changes (FC) are indicated considering the half-sibfamily *g *as the reference group. (two-way ANOVA, *P *< 0.01).

**Table 7:** Genes involved in the main physiological processes regulated by genetic and diet factors interactions.

Physiological process	Swiss prot description	Gene name
Aromatic amino acid family	4-hydroxyphenylpyruvate dioxygenase	HPD
	15-hydroxyprostaglandin dehydrogenase	HPGD
	Peroxisomal multifunctional enzyme type 2	HSD17B4
Nucleotide metabolism	CTP synthase 1	CTPS
	Deoxycytidine kinase	DCK
	GMP reductase 1	GMPR
	5'-nucleotidase	NT5E
	Talin-1	TLN1

In order to validate the accuracy of the microarray data, the *fads2, hmgcr, fabp7, angptl3, cxcl10, gck *and *lpl *genes, which were spotted on the microarray, were also investigated by means of real-time PCR. The comparison of the gene expression pattern obtained through the real-time PCR and microarray approaches, revealed a correlation greater than 0.75 (Table 8).

**Table 8:** Correlation between gene expression patterns obtained through real-time PCR and microarray approaches.

Gene name	Swiss-prot description	Correlation coefficient
*angptl3*	Angiopoietin-related protein 3	0.75
*cxcl10*	C-X-C motif chemokine 10	0.77
*fabp7*	Fatty acid-binding protein	0.86
*gck*	Glycerol kinase	0.90
*hmgcr*	3-hydroxy-3-methylglutaryl-coenzyme A reductase	0.96
*lpl*	Lipoprotein lipase	0.85
*fads2*	Fatty acid desaturase 2	0.89

### Immune parameters

**Figure 1:**
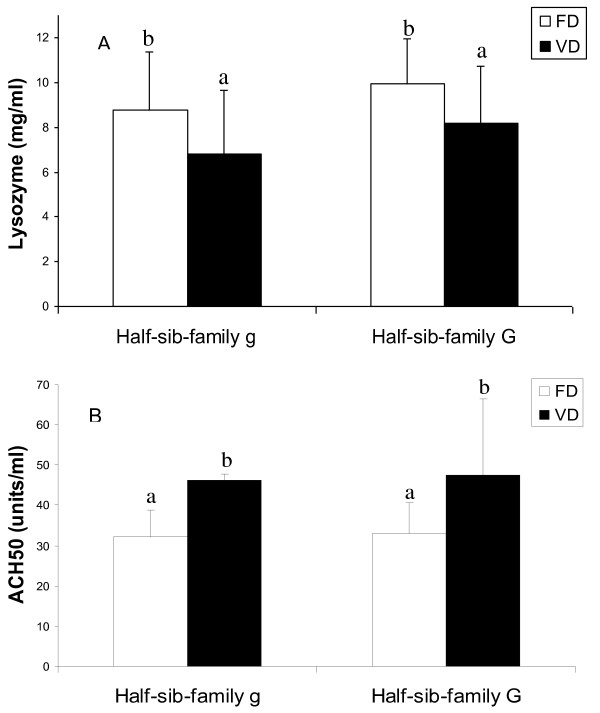
**Influence of the vegetable diet on plasma lysozyme concentration (A) and alternative complement pathway activity (B)**. Results are expressed as mean +/- S.D. (n = 15). Different letters indicate significant differences (two-way ANOVA, *P *< 0.05).

Lysozyme activity was significantly lower (0.7-fold; *P *< 0.05) in fish fed VD than in fish fed FD, while the alternative complement activity was 1.5-times higher (*P *< 0.05) (Figure 1). There was no effect of the half-sibfamily factor on these activities.

## Discussion

The present work is the first investigation into the effect of an exclusively vegetable diet on the hepatic transcriptome in a marine fish species. It is also the first study to have explored the transcriptome of two half-sibfamilies of European sea bass exhibiting different capacities to grow on such a diet. The replacement of FM and FO with increasing levels of plant protein and oil sources for marine fish species can modify feed intake and conversion, which should be the major reason for associated growth delay [37,38]. In the present study, there was a tendency for higher FE in fish fed FD (0.56, 0.60) compared with fish fed VD (0.51, 0.55). However, this difference could not be statistically tested since fish were reared in only two tanks per diet condition. A vegetable diet is also known to potentially impact fish metabolism through regulation of gene expression, especially in the liver [8,39,40]. Analysis of the oligo-DNA microarray data by two-way ANOVA indicated that several hundred genes were differentially regulated according to diet or/and half-sibfamily factors. The accuracy of the present microarray data is validated by the similar gene pattern expression obtained from different oligonucleotides representing the same genes spotted on the array (data not shown), as well as by the correlation shown between results of microarray and qPCR approaches.

### LC-PUFA metabolism

Metabolism-related biological processes constitute the largest group among the GO terms associated with the genes regulated in VD-fed fish. Among these, genes involved in lipid metabolism and, particularly, in LC-PUFA biosynthesis, were found to be up-regulated in fish fed VD. Not surprisingly, delta-6-desaturase (*fads2*), steroyl-CoA desaturase 9 (*scd9*) and NADH-cytochrome b5 reductase (*cyb5r2*), involved in long chain fatty acid desaturation and/or elongation, were up-regulated in VD-fed fish. It is indeed established that in most fish species that the LC-PUFA biosynthetic pathway is positively regulated in response to the use of a diet poor in LC-PUFA but rich in PUFA, although this regulation depends on fish species, degree of fish oil substitution, nature of the vegetable oil, and environmental parameters (e.g., salinity) [41].

The positive regulation of the LC-PUFA biosynthetic pathway is in agreement with results obtained by transcriptomic approaches in salmonids fed vegetable oil [39,40]. Altogether, the expression data obtained in marine fish species and salmonids indicate that the different capacity of marine and fresh water species to grow on a LC-PUFA-deprived diet does not seem to be due to a different transcriptional regulation of key genes involved in lipid synthesis, such as *fads2 *or *scd9*. Indeed, the level of induction of *fads2 *expression found in this experiment (5-fold induction) is of similar amplitude to that observed in the liver and intestine of Atlantic salmon [42].

The stimulation of this biosynthetic pathway in fish fed a diet poor in LC-PUFAs can be explained by the fact that LC-PUFAs play several key physiological roles in vertebrates, particularly in fish. For example, fish are poikilothermic organisms and therefore need a high degree of unsaturation of LC-PUFA included in membrane phospholipids to maintain phospholipid bilayer fluidity at reduced temperature [43]. LC-PUFA, especially arachidonic acid (ARA) and eicosapentaenoic acid (EPA), are also precursors of eicosanoids, which are involved in pro and anti-inflammatory pathways. This hypothesis is reinforced by our data, indicating a stimulation of genes involved in phospholipids biosynthesis (*agpat3*, *pisd *and *pigf*) when fish were fed the VD.

Despite this stimulation of LC-PUFA and the phospholipid biosynthesis pathway at the transcriptional level, our investigation of fatty acid profiles indicated that the amounts of LC-PUFA, particularly eicosapentaenoic acid (EPA) and docosahexaenoic acid (DHA), were still considerably lower in the flesh of fish from both half-sibfamilies fed VD in comparison with fish fed FD. This finding is in agreement with those previously obtained [13], which revealed that the stimulation of *fads2 *expression in fish fed a vegetable diet was not associated with an induction of its enzymatic activity, suggesting a post-transcriptional regulation of *fads2 *expression. Such EPA and DHA deficiency can notably explain the growth deficiency observed in fish fed VD, as well as effects observed on immune function (discussed below).

Concerning the genetic aspect, the comparable overall expression pattern of genes involved in LC-PUFA synthesis in the liver, associated with similar LC-PUFA profile in muscle, in both half-sibfamilies suggests that the differing capacities of these European sea bass half-sibfamilies to grow on a vegetable diet are not due to differing capacities to synthesize LC-PUFA.

### Sterol metabolism

**Figure 2:**
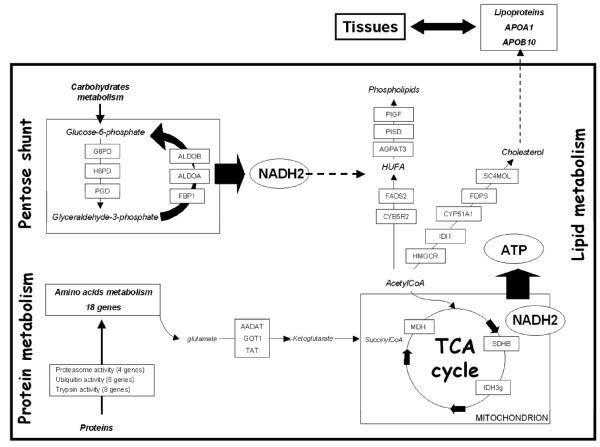
**Main metabolic pathways regulated in the liver of fish fed an all-plant-based diet**. Schematic view of the main metabolic pathways regulated in the liver of European sea bass fed a FM/FO free diet for 9 months. Some major metabolites are indicated in italics. The genes stimulated by the VD in the present study are indicated by their gene name in boxes.

The present microarray data also indicate an increase in expression levels of genes involved in sterol metabolism in VD-fed fish. Among these, isopentenyl-diphosphate delta-isomerase 1 (*idi1)*, lanosterol 14-alpha demethylase *(cyp51a1)*, farnesyl pyrophosphate synthase *(fdps)*, c-4 methylsterol oxidase (*sc4mol*) and *3*-hydroxy-3-methylglutaryl-coenzyme A reductase *(hmgcr) *genes [44-49] are known to be implicated in the cholesterol metabolic pathway (Figure 2). More particularly, HMGCR, a transmembrane glycoprotein involved in the rate-limiting step of sterol biosynthesis, is increased, as shown in mammals [50]. The stimulation of cholesterol biosynthesis in fish fed VD could be related to the difference in sterol composition between diets. Indeed, while the fish diet is rich in cholesterol, the vegetable diet used in this experiment contains exclusively plant sterols, which have been shown to affect membrane properties by decreasing permeability and fluidity, and modifying phospholipid order in mammals [51,52]. As a consequence, the increase in cholesterol biosynthesis could be a metabolic response to its deficiency in the diet, as well as a way to restore membrane properties by incorporation of endogenous cholesterol. Since we did not measure the cholesterol content in the liver, flesh or blood, it is not possible for us to assess the capacity of European sea bass to compensate for possible dietary deficiencies in cholesterol through a regulation of its biosynthesis. Moreover, similarly to the LC-PUFA pathway, no significant difference of cholesterol biosynthetic regulation was observed between the half-sibfamilies.

Interestingly, several VD-stimulated genes (*fads2*, *hmgcr*, *idi1*, *cyp51a1*, *fdps *and *sc4mol*) involved in the lipogenic pathway (LC-PUFA and sterol pathways) are known to be molecular targets of sterol regulatory element binding proteins (SREBPs), which are key regulators of fatty acid and cholesterol synthesis [53,54]. Recent data indicating an up-regulation of the *srebp-1 *gene expression in European sea bass fed a vegetable diet [13] could thus be due to such stimulations.

### Lipid and sterol transport

The present microarray data indicate that the stimulation of genes involved in fatty acid and cholesterol synthesis in VD-fed fish was associated with an over-expression of genes involved in their transport, such as apolipoproteins APOA1 and APOB100, which are the major protein constituents of high and low density lipoprotein (HDL, LDL), respectively (Figure 2). The LDL, including APOB100, are involved in the transport of cholesterol and lipids from the liver to other tissues. Thus, up-regulation of *apob100 *combined with the induction of the expression of lipoprotein lipase (*lpl*), a key enzyme involved in the hydrolysis of triglyceride, suggests an increase in lipid transport and metabolism from the liver to tissues in fish fed VD. The decrease in angiopoietin-related protein 3 (*angptl3*) that we observed in fish fed VD reinforces this idea since ANGPTL3 suppresses LDL clearance via the inhibition of LPL activity [55]. In parallel, we observed that the reverse transport of cholesterol not used by tissues via HDL to the liver was also stimulated in fish fed VD. Indeed, APOA1, which participates in the transport of cholesterol to the liver by promoting cholesterol efflux from tissues and by acting as a cofactor for the lecithin cholesterol acyltransferase (*lcat*), exhibited higher transcript levels in fish fed VD. Altogether these results reveal that another major response in the liver of European sea bass fed a vegetable diet is the stimulation of cholesterol synthesis and transport, irrespective of the half-sibfamily considered.

### Carbohydrate metabolism

LC-PUFA and cholesterol biosynthesis require reducing power in the form of NADPH. It is well documented in vertebrates, including fish, that NADPH required for malonyl-CoA synthesis is mainly supplied by the dehydrogenases of the pentose phosphate shunt [39,56,57]. Interestingly, our transcriptomic data indicate that the use of the VD induced a significant increase in the level of glucose-6-phosphate dehydrogenase (*g6pd *transcripts). G6PD catalyses NADP+-linked oxidation of D-glucose-6-phosphate and has been shown to be a major contributor of NADPH production for lipogenesis in Atlantic salmon (*Salmo salar*) [58] and European sea bass [58]. Moreover, our data indicate an increase in the expression of hexose-6-phosphate dehydrogenase (*h6pdh*) and phosphogluconate dehydrogenase (*pgd*), enzymes of the pentose phosphate pathway that generate NADPH, in fish fed VD [59,60] (Figure 2). Once synthesized, the resulting pentose sugar intermediate generated by the pentose phosphate pathway can be reconverted to intermediates of the glycolysis/gluconeogenesis pathway such as glyceraldehyde 3P or fructose 6P. In the liver of fish, it is known that glycolysis provides essential precursors for biosynthesis rather than pyruvate for oxidation [61]. Thus, the stimulation of fructose-1, 6-bisphosphatase 1 (*fbp1*) and aldolase (*aldoa *and *aldob*) expression that we observe in fish fed VD could provide high levels of fructose-6-phosphate from glyceraldehyde 3P, then glucose 6P that serves as substrate for repeated passage in the pentose phosphate shunt (Figure 2).

### Protein/amino-acid metabolism and ATP synthesis

Our data revealed over-expression of genes involved in proteolysis and, more particularly, in proteasome activity (*psma4, psmb7, psmc2 *and *psmd4*) and ubiquitin activity (*ubap1, ube2a, ube2g1 *and *ube2n*) in fish fed VD (Figure 2), which is in total agreement with proteomic data obtained in rainbow trout, indicating a stimulation of proteolysis in fish fed vegetable diets [8]. In our study, the stimulation of proteolysis in the fish fed the vegetable diet was associated with the induction of 18 genes involved in amino acid metabolism and, more importantly, 4 genes involved in glutamine metabolism. In addition, *gmps*, *aadat*, *got1 *and *tat *genes, which are implicated in transamination, were also stimulated in fish fed VD. The processes related to amino acid metabolism and, especially transamination, are important steps in the synthesis of some non-essential amino acids such as α-ketoglutarate. For example, the synthesis of α-ketoglutarate through transamination reactions could be used in the TCA cycle to provide energy. Interestingly, we found over-expression of genes encoding enzymes involved in the TCA cycle, such as succinate dehydrogenase (*sdhb*), isocitrate dehydrogenase (*idh3g*) and malate dehydrogenase (*mdh*), in fish fed VD. This stimulation of the TCA cycle could be related to the higher levels of ATP required for LC-PUFA and cholesterol biosynthesis in fish fed VD. Since marine fish have a low capacity to digest complex carbohydrates, in contrast to mammals [62,63], the use of proteins as an essential source of energy can thus explain the stimulation of the amino acid metabolism in fish fed VD. As shown in rainbow trout fed on a vegetable-based diet [64], the lower growth rate in fish fed VD in the present study could be associated with higher proteolytic activity compared with fish fed FD.

Interestingly, while both half-sibfamilies *G *and *g *exhibited similar proteolysis regulation, the expression of several genes involved in macromolecule biosynthesis, and particularly in protein biosynthesis (*cct2, eif4g1, kiaa2018, mrpl22, mrpl27, mrpl30, mrpl34, mrpl48, mrps14, mrps17, rars, rpl18, rplp1 *and *rps18*), were up-regulated in half-sibfamily *G*. This result, suggesting a higher protein turnover in half-sibfamily *G *compared with half-sibfamily *g *when fish were fed VD, could be related to the higher growth rate observed in half-sibfamily *G *fed VD. As protein biosynthesis requires energy from ATP hydrolysis, the higher protein biosynthesis in half-sibfamily could be related to a higher activity of mitochondrial ATP production. Accordingly, genes involved in ATP biosynthesis (*atp5c1, atp5i *and *atp5j2*) and ATP synthesis-coupled electron transport (*ndufb4, ndufb6, ndufs4, ndufs6, ndufv2 *and *uqcrb*) were found up-regulated in half-sibfamily *G*. The diet × half-sibfamily interaction that we found for the expression of genes involved in aromatic amino acid metabolism reinforces the difference in protein metabolism between the two half-sibfamilies.

### Immune function

In the present study, the vegetable diet used, based on linseed oil, was characterised by a very low ARA content and poor levels of n-3 LC-PUFA. Eicosanoids derived from ARA are known to be involved in the proliferation of hepatocytes and immune cells [65-67]. As a consequence, the lower hepatosomatic index (HSI) measured in fish fed VD could be linked to a lower hepatocyte proliferation due to a deficiency in ARA, which was supported by down-expression of 32 genes involved in cell proliferation in this dietary group. Moreover, fatty acid imbalances can induce an immune deficiency in all vertebrates including fish [14,15,22]. In particular, the Σ n-3/Σ n-6 fatty acid ratio is considered as a key element regulating immune cell structure, cell signalling, and eicosanoid production.

The present microarray data revealed genes of the immune system, particularly the innate immune response, exhibiting lower expression in fish fed with VD. Interleukin 8 (*il8*) and C-X-C motif chemokine 10 (*cxcl10*), which are chemotactic factors for granulocytes and monocytes, respectively, were found to be less expressed in fish fed VD, suggesting a down regulation of the innate defence system and the pro-inflammatory pathway in this group. Deficiency of the inflammatory response was also in agreement with the higher levels of transcripts of fatty acid binding protein 7 (*fabp7*), whose expression in mammals has been shown to be restricted to the Kupffer cells [68], and the down expression of the C-reactive protein (*crp*), an acute phase protein synthesised by hepatocytes, in fish fed VD. Decrease in inflammatory response can also be related to the low level of ARA in the fish fed VD, which induces a reduction of prostaglandin synthesis derived from this fatty acid. Our microarray data indeed show that prostaglandin E synthase 2 (*ptgs2*), involved in the synthesis of pro-inflammatory prostaglandin E2, is down-regulated in fish fed VD, while prostaglandin E synthase 3 (*ptgs3*), which has anti-inflammatory properties, exhibited higher messenger levels in fish fed VD. This depression of innate immune system, particularly pro-inflammatory activity, could also be partially explained by a defect in membrane properties in fish fed VD, as revealed by the down-regulation of a large number of genes (65) related to cell communication, including factors such as cytokine receptor common subunit gamma (*il2rg*), receptor-type tyrosine-protein phosphatase F (*ptprf*) or integrin beta 2 (*itgb2*), which are cell-surface receptor binding proteins and/or cell adhesion receptors involved in immune response. The depression of the innate immune response in fish fed VD was confirmed by the lower plasmatic lysozyme concentration and lower-expression of lysozyme g (*lyg*) gene. Surprisingly, the alternative complement pathway activity involved in the innate immune response, which we assessed by analysis of plasma parameters, showed a significantly higher level in fish fed VD. Such an opposite regulation of the immune pathway revealed that different components of the immune systems can be regulated in opposite directions.

Interestingly, processes related to the humoral immune response were also over-represented among the genes up-regulated in half-sibfamily *g*. Indeed, *complement component c2, c3 *and *c9 *genes showed higher expression levels in half-sibfamily *g*. However, the up-regulation of genes involved in the alternative complement pathway cannot be associated with an increase of the plasma alternative complement pathway activity, probably due to the complexity of factors and regulation levels (transcriptional and post-transcriptional) involved in the regulation of this pathway [69]. Moreover, the higher expression of *masp2*, *tnrfrf14*, *c2 *and *c3 *genes involved in the inflammatory response might reflect higher inflammatory states in half-sibfamily *g*, which could be associated with a decrease in growth rate, as demonstrated in chicken [70].

### Blood coagulation

**Figure 3:**
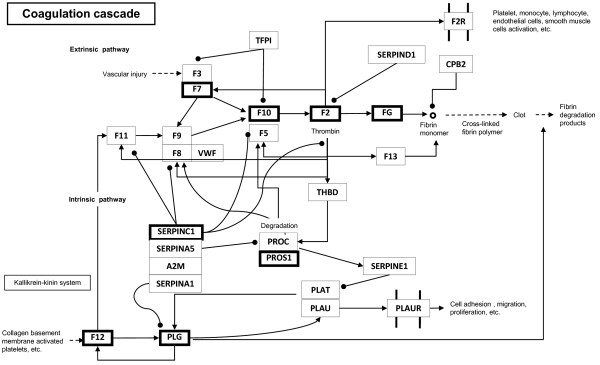
**Genes involved in the coagulation process that are regulated by the diet composition (FD/VD)**. The mammalian coagulation cascade pathway is shown. Pathway information is adapted from the KEGG database [74]. Genes significantly stimulated by the use of the VD are indicated in bold in boxes (*serpinc1*, *pros1*, *plg*, *f12*, *f7*, *f10*, *fgb *(*fg*) and *fgg *(*fg*)).

Blood coagulation is another process involved in the innate immune system. LC-PUFA and, more specifically, EPA, DHA and ARA are precursors for eicosanoid synthesis involved in the control of the blood coagulation [71-73]. As mentioned above, the use of a diet composed of vegetable protein and oil induces modifications in the membrane phospholipid composition, with possible consequences for eicosanoid production and the blood coagulation process. We found that the use of the vegetable diet induced an increase in the expression of genes involved in the blood coagulation pathway (Figure 3). Among these genes, prothrombin (*f7*), coagulation factor × (*f10*), fibrinogen beta chain (*fgb*), fibrinogen gamma chain (*fgg*) and coagulation factor VII (*f7*) were positively involved in the blood coagulation process. On the basis of these results, the use of a VD seems to cause pro-coagulant action by the stimulation of the blood coagulation pathway, which is in agreement with our visual observation of plasma clotting (data not shown). In agreement with these results, Tavares-Dias [23] showed that, contrary to a vegetable diet, dietary enrichment in long-chain n-3 fatty acids has a strong hypocoagulant action. In addition, the regulation of the coagulation pathway is complex and under the control of several negative factors that maintain a physiological homeostasis. The pro-coagulation effect in response to a vegetable diet is notably reinforced by some of these genes [the vitamin K-dependent protein S (*pros1*), plasminogen (*plg*) and antithrombin-III (*serpinc1*)], which also exhibited higher expression in fish fed VD (Figure 3).

## Conclusions

The nutrigenomic approach used here has revealed several new genes and related biological processes regulated by a vegetable diet. In particular, genes involved in lipid metabolism, protein/amino acid metabolism, carbohydrate metabolism, immune function, blood coagulation and the RNA splicing process were expressed at a higher level in fish fed with VD. The comparison of transcriptomic response in two half-sibfamilies of fish exhibiting different growth rates when fed the vegetable diet also revealed some biological processes related to protein turnover and immune response, potentially due to better adaptation to this diet. Finally, in the context of developing novel diets for aquaculture and selecting fish families exhibiting higher adaptation to fish oil and meal substitution, this work enabled us to pinpoint potentially useful new molecular markers for identifying the physiological effects of a vegetable diet, as well as a family exhibiting a phenotype of interest.

## List of abbreviations

ALA: α-linolenic acid; ARA: arachidonic acid; DGC: daily growth coefficient; DHA: docosahexaenoic acid; DM: dry matter; EF1: elongation factor 1; EPA: eicosapentaenoic acid; FD: fish diet; FE: feed efficiency; GO: gene ontology; HDL: high density lipoprotein; HSI: hepatosomaticindex; LA: linoleic acid; LC-PUFA: long chain polyunsaturated fatty acid; LDL: low density lipoprotein; NL: neutral lipid; PL: polar lipid; PUFA: polyunsaturated fatty acid; TL: total lipid; VD: vegetable diet; VSI: viscerosomatic index.

## Authors' contributions

FG and DM co-ordinated the sampling procedures, biochemical studies and microarray data analysis, and drafted the manuscript. SF and LB carried out the sample preparation, hybridizations and microarray data normalizations. SK formulated the experimental diet. MV participated in the design of the study and performed analysis of growth parameters. CC and JLZ conceived and designed the study and led its coordination. CQ co-ordinated dedicated experiments on immune function. All authors read and approved the final manuscript.

## Supplementary Material

Additional file 1 **Comparison of Ct values for *ef1 *gene between the dietary groups**. Quantitative PCR did not reveal any significant difference (t-student test, p > 0. 1) between Ct values for Ef1 between the dietary groups (FD: Fish diet; VD: Vegetable diet; G: G-sibfamily; g: g-sibfamily). Click here for file

Additional file 2 **Fatty acid composition in muscle of two European sea bass half-sibfamilies fed FD or VD**. Composition in terms of the main fatty acids (% of total fatty acids) in neutral lipid and phospholipid fractions in muscle of each of the half-sibfamilies (*g *and *G*) of European sea bass fed FD or VD. Effects of diet factor and half-sibfamily factor on fatty acid composition were determined by two-way ANOVA. Results are expressed as mean +/- S.D. (n = 15) and significant differences are indicated by the *p *value (two-way ANOVA, *P *< 0.05). Click here for file

Additional file 3 **Significantly enriched biological processes associated with genes regulated by diet and half-sibfamily factors (EASE, *P *< 0.05)**. The main biological processes enriched out of those associated with genes that were over-expressed in fish fed VD were related to physiological process, metabolism (sterol metabolism, carboxylic metabolism, amino acid metabolism), RNA splicing, protein catabolism, aerobic respiration, blood coagulation and hexose catabolism. In contrast, the main biological processes associated with the genes lower-expressed in fish fed VD were related to cellular process, cell communication and cell proliferation. Regarding half-sibfamily factors, biological process related to Humoral immune response was shown to be over-represented within genes up-expressed in half-sibfamily *g *while processes related to energy pathways (ATP synthesis, mitochondrial electron transport) were enriched within genes up-expressed in half-sibfamily *G*. Click here for file
