# Supplementary material for: Effects of the total replacement of fish-based diet with plant-based diet on the hepatic transcriptome of two European sea bass (Dicentrarchus labrax) half-sibfamilies showing different growth rates with the plant-based diet
Source: BMC Genomics. 2011 Oct 23;12:522. doi: 10.1186/1471-2164-12-522 (PMC3377934; doi:10.1186/1471-2164-12-522)
Supplement: Additional file 2 — Fatty acid composition in muscle of two European sea bass half-sibfamilies fed FD or VD. Composition in terms of the main fatty acids (% of total fatty acids) in neutral lipid and phospholipid fractions in muscle of each of the half-sibfamilies (g and G) of European sea bass fed FD or VD. Effects of diet factor and half-sibfamily factor on fatty acid composition were determined by two-way ANOVA. Results are expressed as mean +/- S.D. (n = 15) and significant differences are indicated by the p value (two-way ANOVA, P < 0.05). [file 1471-2164-12-522-S2.DOC]

Additional file 2

|  | **FD** | |  | **VD** | |  |  |  |  |  |  |
| --- | --- | --- | --- | --- | --- | --- | --- | --- | --- | --- | --- |
|  | **HSF g** | **HSF G** |  | **HSF g** | **HSF G** |  | **Diet factor** |  | **Sib family factor** |  | **Diet X Sib family factors** |
| **Neutral lipds** |  |  |  |  |  |  | *p* value |  | *p* value |  | *p* value |
| LN (%DM) | 6.4 ± 3.8 | 6.2 ± 3.6 |  | 7.6 ± 3.1 | 5.5 ± 2.0 |  | NS |  | NS |  | NS |
| Σ saturates | 34.5 ± 1.8 | 34.4 ± 3.4 |  | 23.6 ± 2.2 | 22.6 ± 2.5 |  | 0.0001 |  | NS |  | NS |
| Σ monoenes | 33.0 ± 7.2 | 30.4 ± 8.3 |  | 30.2 ± 4.5 | 17.8 ± 1.0 |  | NS |  | NS |  | NS |
| 18:2n-6 | 6.7 ± 2.5 | 5.8 ± 1.1 |  | 14.9 ± 2.5 | 15.9 ± 1.0 |  | 0.0001 |  | NS |  | NS |
| 20:2n-6 | 0.4 ± 0.2 | 0.4 ± 0.1 |  | 0.8 ± 0.3 | 1.0 ± 0.1 |  | 0.0001 |  | NS |  | 0.0089 |
| 18:3n-6 | 0.0 ± 0.0 | 0.0 ± 0.0 |  | 0.0 ± 0.0 | 0.0 ± 0.0 |  | - |  | - |  | - |
| 20:3n-6 | 0.1 ± 0.0 | 0.1 ± 0.0 |  | 0.0 ± 0.0 | 0.1 ± 0.1 |  | NS |  | NS |  | NS |
| 20:4n-6 | 0.6 ± 0.5 | 0.7 ± 0.6 |  | 0.1 ± 0.1 | 0.2 ± 0.3 |  | 0.0001 |  | NS |  | NS |
| Σ n-6 PUFA | 7.8 ± 0.5 | 7.0 ± 1.5 |  | 15.8 ± 0.4 | 17.2 ± 0.4 |  | 0.0001 |  | NS |  | 0.0213 |
| 18:3n-3 | 2.3 ± 4.7 | 0.9 ± 0.2 |  | 18.2 ± 3.2 | 19.1 ± 2.4 |  | 0.0001 |  | NS |  | NS |
| 18:4n-3 | 0.9 ± 0.2 | 0.9 ± 0.3 |  | 1.0 ± 0.3 | 1.0 ± 0.2 |  | NS |  | NS |  | NS |
| 20:3n-3 | 0.1 ± 0.1 | 0.0 ± 0.0 |  | 0.2 ± 0.1 | 0.3 ± 0.1 |  | 0.0001 |  | 0.0451 |  | 0.0028 |
| 20:4n-3 | 0.5 ± 0.1 | 0.5 ± 0.1 |  | 0.2 ± 0.1 | 0.2 ± 0.0 |  | 0.0001 |  | NS |  | NS |
| 20:5n-3 | 6.5 ± 2.0 | 7.0 ± 1.6 |  | 2.2 ± 1.6 | 2.0 ± 0.8 |  | 0.0001 |  | NS |  | NS |
| 22:5n-3 | 1.1 ± 0.3 | 1.3 ± 0.3 |  | 0.3 ± 0.3 | 0.3 ± 0.1 |  | 0.0001 |  | NS |  | NS |
| 22:6n-3 | 8.2 ± 4.1 | 10.3 ± 5.3 |  | 1.9 ± 2.3 | 2.5 ± 2.6 |  | 0.0001 |  | NS |  | NS |
| Σ n-3 PUFA | 19.1 ± 1.9 | 20.9 ± 2.1 |  | 24.0 ± 1.1 | 25.4 ± 1.2 |  | 0.0004 |  | NS |  | NS |
| **Phospholipids** |  |  |  |  |  |  |  |  |  |  |  |
| PL (%DM) | 5.7 ± 2.6 | 5.0 ± 0.8 |  | 4.0 ± 1.2 | 4.2 ± 1.2 |  | 0.0057 |  | NS |  | NS |
| Σ saturates | 37.3 ± 3.9 | 35.7 ± 1.9 |  | 29.4 ± 2.7 | 27.6 ± 1.8 |  | 0.0001 |  | NS |  | NS |
| Σ monoenes | 17.8 ± 1.4 | 23.0 ± 2.5 |  | 19.4 ± 1.1 | 20.1 ± 4.4 |  | NS |  | NS |  | NS |
| 18:2n-6 | 4.0 ± 3.3 | 3.5 ± 1.0 |  | 15.8 ± 0.9 | 17.8 ± 1.1 |  | 0.0001 |  | NS |  | NS |
| 20:2n-6 | 0.2 ± 0.1 | 0.3 ± 0.1 |  | 0.7 ± 0.2 | 0.9 ± 0.1 |  | 0.0001 |  | 0.0105 |  | NS |
| 18:3n-6 | 0.0 ± 0.0 | 0.0 ± 0.0 |  | 0.0 ± 0.0 | 0.0 ± 0.0 |  | - |  | - |  | - |
| 20:3n-6 | 0.1 ± 0.1 | 0.1 ± 0.1 |  | 0.2 ± 0.1 | 0.3 ± 0.1 |  | 0.0001 |  | NS |  | NS |
| 20:4n-6 | 2.3 ± 0.4 | 2.1 ± 0.8 |  | 1.1 ± 0.4 | 0.8 ± 0.3 |  | 0.0001 |  | NS |  | NS |
| Σ n-6 PUFA | 6.6 ± 0.3 | 5.9 ± 0.4 |  | 17.8 ± 0.5 | 19.8 ± 0.5 |  | 0.0001 |  | NS |  | 0.0391 |
| 18:3n-3 | 1.2 ± 3.3 | 0.4 ± 0.2 |  | 13.2 ± 3.9 | 15.7 ± 1.9 |  | 0.0001 |  | NS |  | 0.0317 |
| 18:4n-3 | 0.2 ± 0.1 | 0.3 ± 0.2 |  | 0.5 ± 0.1 | 0.6 ± 0.2 |  | 0.0001 |  | 0.0423 |  | NS |
| 20:3n-3 | 0.0 ± 0.0 | 0.0 ± 0.0 |  | 0.2 ± 0.1 | 0.3 ± 0.0 |  | 0.0001 |  | 0.0209 |  | 0.0004 |
| 20:4n-3 | 0.3 ± 0.1 | 0.3 ± 0.0 |  | 0.1 ± 0.1 | 0.2 ± 0.1 |  | 0.0001 |  | 0.0154 |  | NS |
| 20:5n-3 | 11.5 ± 1.7 | 10.6 ± 2.7 |  | 4.8 ± 2.3 | 3.3 ± 0.8 |  | 0.0001 |  | 0.0274 |  | NS |
| 22:5n-3 | 1.2 ± 0.2 | 1.3 ± 0.2 |  | 0.7 ± 0.2 | 0.6 ± 0.2 |  | 0.0001 |  | NS |  | NS |
| 22:6n-3 | 23.3 ± 4.6 | 22.8 ± 4.3 |  | 10.4 ± 4.4 | 8.4 ± 3.3 |  | 0.0001 |  | NS |  | NS |
| Σ n-3 PUFA | 37.4 ± 1.8 | 35.7 ± 1.3 |  | 29.2 ± 1.5 | 29.1 ± 1.1 |  | 0.0015 |  | NS |  | NS |
