# Supplementary material for: Effects of the total replacement of fish-based diet with plant-based diet on the hepatic transcriptome of two European sea bass (Dicentrarchus labrax) half-sibfamilies showing different growth rates with the plant-based diet
Source: BMC Genomics. 2011 Oct 23;12:522. doi: 10.1186/1471-2164-12-522 (PMC3377934; doi:10.1186/1471-2164-12-522)
Supplement: Additional file 3 — Significantly enriched biological processes associated with genes regulated by diet and half-sibfamily factors (EASE, P < 0.05). The main biological processes enriched out of those associated with genes that were over-expressed in fish fed VD were related to physiological process, metabolism (sterol metabolism, carboxylic metabolism, amino acid metabolism), RNA splicing, protein catabolism, aerobic respiration, blood coagulation and hexose catabolism. In contrast, the main biological processes associated with the genes lower-expressed in fish fed VD were related to cellular process, cell communication and cell proliferation. Regarding half-sibfamily factors, biological process related to Humoral immune response was shown to be over-represented within genes up-expressed in half-sibfamily g while processes related to energy pathways (ATP synthesis, mitochondrial electron transport) were enriched within genes up-expressed in half-sibfamily G. [file 1471-2164-12-522-S3.DOC]

Additional file 3 :

|  | **GO biological process category** | Genes with GO term in input list | Genes with GO in output list | Enrichment p-Value |
| --- | --- | --- | --- | --- |
|  | Physiological process | 2683 | 324 | 6.7 x 10^-3^ |
|  | Metabolism | 1942 | 267 | 2.0 x 10^-7^ |
|  | RNA splicing | 48 | 16 | 1.8 x 10^-4^ |
|  | Protein catabolism | 189 | 34 | 7.6 x 10^-3^ |
|  | Aerobic respiration | 8 | 5 | 8.1 x 10^-3^ |
|  | Sterol metabolism | 23 | 8 | 0.01 |
| **Enriched GO associated with the genes** | Carboxylic acid metabolism | 169 | 30 | 0.01 |
| **over expressed in fish fed VD** | Amino acid biosynthesis | 20 | 7 | 0.02 |
|  | Blood coagulation | 51 | 12 | 0.03 |
|  | Ribosome biogenesis | 22 | 7 | 0.04 |
|  | Glutamine metabolism | 7 | 4 | 0.04 |
|  | Amino acid catabolism | 23 | 7 | 0.04 |
|  | Homostasis | 55 | 12 | 0.04 |
|  | Hexose catabolism | 31 | 8 | 0.05 |
|  | Cellular process | 1604 | 136 | 3.3 x 10^-3^ |
| **Enriched GO associated with the genes** | Skeletal development | 43 | 9 | 0.01 |
| **over expressed in fish fed FD** | Cell communication | 734 | 65 | 0.04 |
|  | Cell proliferation | 316 | 32 | 0.04 |
|  | Synaptic transmission | 47 | 8 | 0.05 |
| **Enriched GO associated with the genes** | Humoral immune response | 47 | 5 | 0.03 |
| **over expressed in fish of half-sib-family *g*** |  |  |  |  |
| **Enriched GO associated with the genes** | ATP synthesis couple electron | 17 | 6 | 2.7 x 10^-4^ |
| **over expressed in fish of half-sib-family G** | Mitochondrial electron transport | 15 | 5 | 1.7 x 10^-3^ |
|  | Energy pathways | 108 | 9 | 0.04 |
| **Enriched GO associated with the genes** | aromatic amino acid family metabolism | 12 | 3 | 0.03 |
| **regulated by diet and genetic factors** | nucleotide metabolism | 57 | 5 | 0.04 |
